# Heterogeneity of the definition of elderly age in current orthopaedic research

**DOI:** 10.1186/s40064-015-1307-x

**Published:** 2015-09-17

**Authors:** Sanjeeve Sabharwal, Helen Wilson, Peter Reilly, Chinmay M. Gupte

**Affiliations:** Department of Trauma and Orthopaedics, St Mary’s Hospital, Ground Floor, Salton House, South Wharf Road, London, W2 1NY UK; Department of Geriatrics, Royal Surrey County Hospital NHS Foundation Trust, Egerton Road, Guilford, GU2 7XX UK

**Keywords:** Orthopaedics, Elderly age, Methodology, Frailty

## Abstract

Medical research often defines a person as elderly when they are 65 years of age or above, however defining elderly age by chronology alone has its limitations. Moreover, potential variability in definitions of elderly age can make interpretation of the collective body of evidence within a particular field of research confusing. Our research goals were to (1) evaluate published orthopaedic research and determine whether there is variability in proposed definitions of an elderly person, and (2) to determine whether variability exists within the important research sub-group of hip fractures. A defined search protocol was used within PubMed, EMBASE and the Cochrane Library that identified orthopaedic research articles published in 2012 that stated within their objective, intent to examine an intervention in an elderly population. 80 studies that included 271,470 patients were identified and subject to analysis. Four (5 %) studies failed to define their elderly population. The remaining 76 (95 %) studies all defined elderly age by chronology alone. Definitions of an elderly person ranged from 50 to 80 years and above. The most commonly used age to define an elderly person was 65, however this accounted for only 38 (47.5 %) of studies. Orthopedic research appears to favor defining elderly age by chronology alone, and there is considerable heterogeneity amongst these definitions. This may confuse interpretation of the evidence base in areas of orthopaedic research that focus on elderly patients. The findings of this study underline the importance of future research in orthopaedics adopting validated frailty index measures so that population descriptions in older patients are more uniform and clinically relevant.

## Background

The World Aging Report published by United Nations in 2013 stated that population aging is unprecedented, enduring and has profound global socio-economic implications (United Nations 2013, Department of Social Affairs, Population). The impact of an older population demographic on healthcare spending can be seen in the United Kingdom’s National Health Service (NHS), where between 2007 and 2008, the average value for NHS services for retired households was £5200 compared to £2800 for non-retired households (Cracknell 2010). In the past, archaic medical beliefs often meant that healthcare professionals held negative attitudes towards caring for elderly patients, however in the twenty first century ageist attitudes are increasingly challenged (Lovell 2006), and there is growing awareness of the need for clinical research and treatment focused on the elderly population (Hempenius et al. 2013).

Persisting deficiencies in the care received by elderly patients underline the need for an improving standard of care (Wilkinson 2010). Research focus on treatment interventions in the elderly may relate to differences in clinical outcome as well healthcare expenditure compared to a younger population (Hamel et al. 2005; Yang et al. 2003). Although mortality rates inevitably increase with advancing age (Yang et al. 2003), clinical research has delivered improved clinical outcomes for these patients across a wide range of medical sub-specialties (Nishihata et al. 2013; Partridge et al. 2014). In orthopaedic surgery, focus on an ageing population is especially relevant owing to the association of advanced age with chronic musculoskeletal conditions, such as osteoarthritis, as well as an increased incidence of fragility fractures (Woolf and Pfleger 2003). Furthermore, an evolving understanding of how age related loss of muscle mass and function, or sarcopenia, correlates with conditions such as osteoporosis draws attention to the importance of physiological measures of ageing in musculoskeletal medicine (Matthews et al. 2011).

Although many countries consider a person to be elderly when they have reached 65 years of age, this view is often disputed because of improving life expectancy, quality of life and level of function within an aged population (Sanderson and Scherbov 2008). Some organisations suggest using the age at which entitlement to state pensions commences, however definitions of an elderly population are multidimensional and often accommodate factors such as chronology, change in social role and change of capabilities (United Nations 2012). The complexity of defining elderly age means that examining the treatment benefits in an aged population has the potential to be challenging to clinicians practicing evidence based medicine. The key issue is whether the definition of an elderly person is uniform when examining specific areas of clinical practice, or whether it is variable, and hence produces uncertainty in interpreting the available body of evidence.

The primary objective of this survey study was to evaluate published orthopaedic research and determine what the proposed definitions of an elderly person were, and whether there was variability in the proposed definitions. The secondary objective of this study was to perform sub-group analysis within the field of hip fracture research. This area of orthopaedics deals primarily with older patients with high mortality rates, and therefore a well-defined population is key to clinicians applying the findings of research effectively and appropriately to their practice.

## Methods

### Review protocol

As there is limited guidance on the performance of survey-based research, this study was performed in accordance with the guidelines from the preferred reporting items for systematic reviews and meta-analyses (PRISMA) (Moher et al. 1999).

### Information source and search strategy

**Fig. 1:**
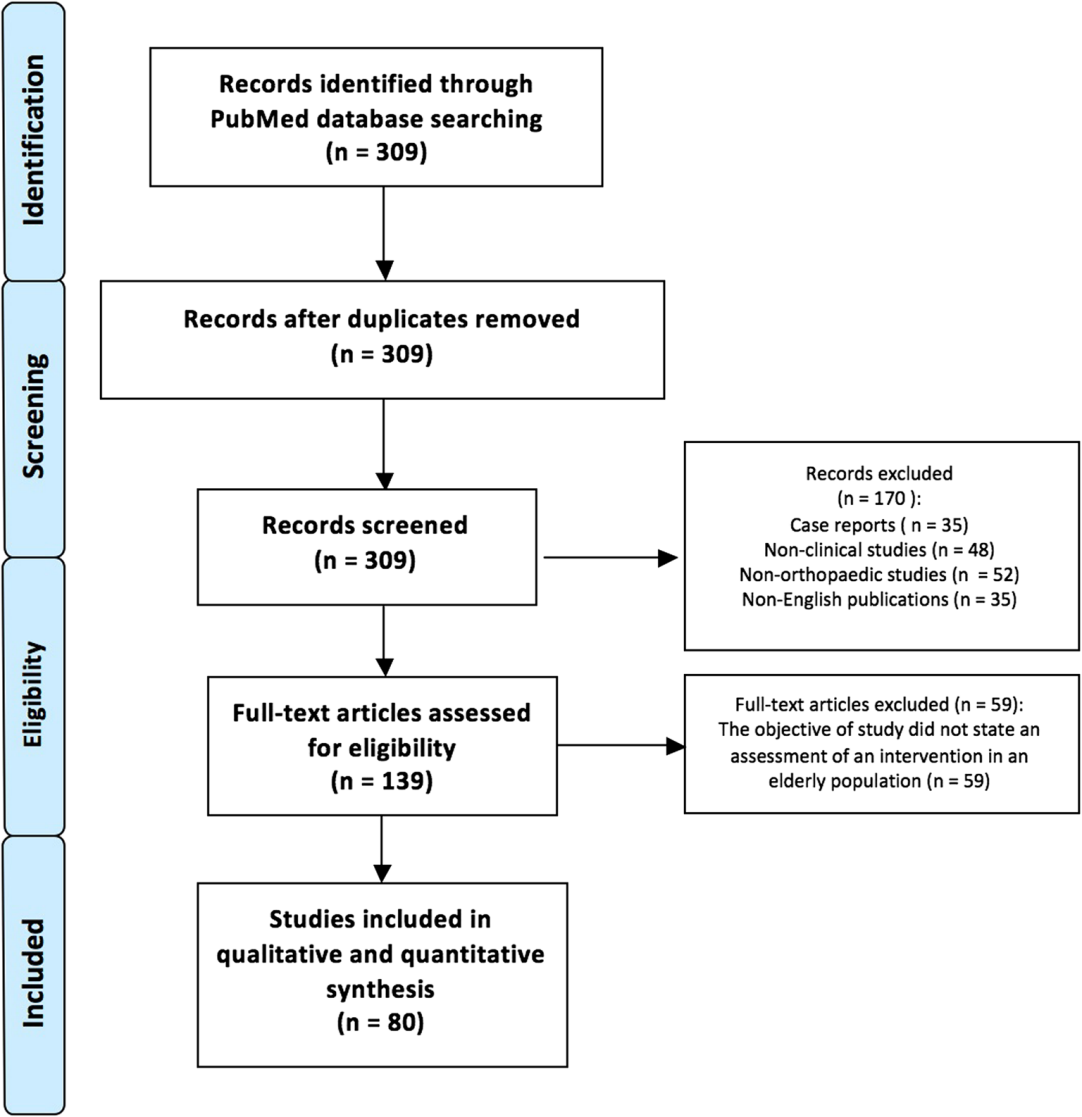
Flow diagram showing systematic search strategy for study selection

A literature search was performed in PubMed, EMBASE and the Cochrane Library for orthopaedic research studies published in 2012. An advanced search was performed using the words “orthopaedic” and “elderly”. The last date of the search was 25th August 2014. A summary of the search strategy is summarized in Fig. 1.

### Inclusion and exclusion criteria

Only clinical research articles that stated within their objective intent to examine an orthopaedic intervention in an elderly population were evaluated. Studies that were published online in 2012 ahead of their print editions were included. Case reports, editorials and correspondence articles were excluded. Articles that were not published in English were also excluded. Furthermore, animal and laboratory studies were excluded from the analysis.

### Data extraction

The following information was obtained from each study: the study region, the area of orthopaedic sub-specialty, level of evidence of the paper according to Oxford Centre for Evidence based Medicine, what the definition of elderly age was stated to be, whether an age range had been provided to define an elderly person, what that age range was and finally, if the study had evaluated the management of patients with a hip fracture.

### Statistical analysis

Descriptive statistics were produced and data were analyzed in SPSS 20.0 (SPSS Inc, Chicago, IL). The distribution of the data was assessed with a Kolmogorov–Smirnov test and found to be non-parametric. Comparison of ages used to define an elderly person between the various studies based on their area of sub-specialty, region of development and level of evidence were performed using a Kruskal–Wallis one-way analysis of variance. Comparison of variance of definitions of elderly patients between hip fracture studies and all other clinical studies was performed using a Mann–Whitney test. A *p* value < 0.05 was considered statistically significant.

## Results

**Fig. 2:**
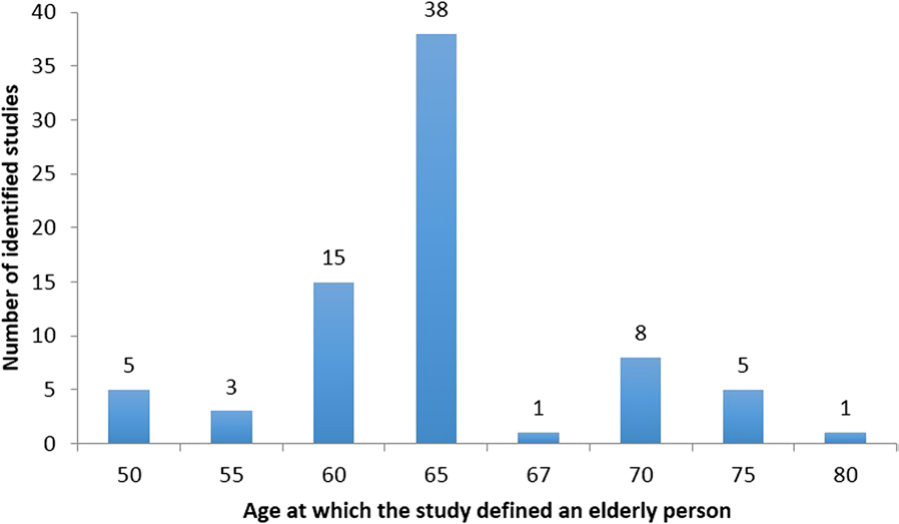
Column chart showing variability in definitions of elderly population according to chronological age

There were 80 studies identified using the search strategy that met the eligibility criteria and were subject to analysis in this study. A total of 271,470 patients were included in the selected orthopaedic research. A total of 76 (95 %) of studies defined elderly age in relation to chronological age alone. There were 4 (5 %) studies that stated within their objectives an intention to examine an intervention within an elderly population, however failed to explain what their definition of an elderly person was in the methodology. Demographic description of the research in relation to region of development, orthopaedic sub-specialty and level of evidence is shown in Table 1. The chronological definitions of an elderly person are shown in Fig. 2. The most commonly used age to define an elderly person was 65 and this was found in 38 (47.5 %) of studies.

**Table 1:** Demographic description of the orthopaedic research in relation to region of development, orthopaedic sub-specialty and level of evidence

	Median age	Range	Standard deviation	Variance
Development region				
North America (n = 18)	65	50–70	4.93	24.27
Europe (n = 19)	65	50–75	6.39	40.81
Asia (n = 41)	65	50–80	6.29	39.62
South America (n = 1)	65	–	–	–
Africa (n = 1)	65	–	–	–
Sub-specialty				
Trauma (n = 45)	65	50–80	5.88	34.61
Upper limb (n = 4)	62.5	50–65	7.07	50
Pelvis/hip/knees (n = 11)	65	50–75	6.71	50
Spine (n = 19)	65	50–75	5.52	30.52
Foot and ankle (n = 1)	70	–	–	–
Level of evidence				
I	65	60–67	3.61	13
II	65	60–75	5.48	30
III	65	50–80	5.96	35.56
IV	62.5	50–75	6.21	38.64

A range of 50–80 years across all studies revealed lack of uniformity in definitions of an elderly person. Moreover this variation was found when individual studies were examined according to their selected demographics. A Kruskal–Wallis test comparing the definitions of age between different regions of development found all groups originated from the same sample distribution (*p* = 0.43). Similarly, assessment of definitions of age by orthopaedic subspecialty (*p* = 0.68) and level of evidence of the studies (*p* = 0.28) using the Kruskal–Wallis test did not demonstrate any significant difference between grouping samples.

There were 26 hip fracture studies identified within the study sample. Two of these papers failed to define the elderly population that they set out to study. Within the remaining 24 articles, 14 (58.30 %) used the age 65 to define an elderly population (Table 2). Although this appeared to compare favorably to non-hip fracture studies of which 24 of 52 (46.15 %) referenced 65 as a definition for an elderly person, comparison of the two groups using a Mann–Whitney test found no significant difference between the two populations (*p* = 0.158).

**Table 2:** Sub-group analysis comparing hip fracture and non-hip fracture studies’ definitions of elderly age

	Median age	Range	Standard deviation	Variance
Hip fracture studies (n = 24)	65	50–80	6.08	36.83
Non hip fracture studies (n = 52)	65	50–75	5.90	34.83
Comparison of data samples with a two tailed Man Whitney test	*p* = 0.158			

Non-parametric analysis between the two groups reveals no significant difference between the two populations (*p* > 0.05). There were two studies in each group that are excluded from the results displayed in this table that failed to define the elderly population they were studying

## Discussion

This study has demonstrated that orthopaedic research favors defining elderly age by chronological age measures alone, and there is variation between these proposed values. Although 65 years of age is the most common definition at which a person is considered to be elderly, this accounted for less than half (47.50 %) of the studies that were examined. Inconsistencies in definitions of age were not restricted to certain study regions, orthopaedic sub-specialties or the level of evidence. Moreover sub-group analysis of hip fracture studies revealed that although there was more consistency in proposed definitions than amongst general orthopaedics studies, even within a field of orthopaedic research that has a strong focus on an aged and vulnerable population, there is a lack of uniformity in defining an elderly person.

The importance of systematic methodology is repeatedly underlined in orthopaedic research as a result of existing evidence that suggests ongoing failings of research quality and reporting (Chess and Gagnier 2013). Although guidance exists for the design and reporting of randomized controlled trials (Schulz et al. 2010), meta-analysis (Moher et al. 2009) and observational studies (Vandenbroucke et al. 2007), uptake and endorsement of such guidance is still not common place in medical journals (Turner et al. 2012). Such tools include within their guidance on methodology a framework for appropriately defining a population, however, definitions of an elderly population are absent from existing guidance.

The elderly are often defined as persons aged 65 years or older (Crews and Zavotka 2006). In countries with advanced economies this sub-group is increasing rapidly and accounts for almost 15 % of their population (Crews 2005). As the population of aged citizens grows, societal and economic pressures to care for them grows proportionally (Crews 2005). Despite this, a large proportion of people over the age of 65 are healthy and live independently (Crews and Zavotka 2006) Consequently, the definition of elderly age by chronology in medical research or health economic evaluations may have its limitations. Firstly, there are demographic variations amongst definitions of age by chronology. Although the World Health Organisation defines patients as elderly if they are 65 years or older, owing to differences in socio-economic conditions and life expectancy, for the purpose of their population studies in Africa a person is defined as elderly if they are 50 years or older (United Nations 2012). Secondly, as life expectancy and population health improve with advances in medicine, an age defined elderly population in the twenty first century may be physiologically healthier and functionally more capable that those in the twentieth century (Sanderson and Scherbov 2008; Crews and Zavotka 2006). Such considerations mean that defining elderly age by chronology in medical research could produce inconsistent definitions that are not relevant to the purpose of the research objective. The variability of reported ages to define an elderly person in this study underline the need for evidence based methods of defining an older population in orthopaedic research.

The concept of frailty in elderly patients is long established in clinical medicine, however has considerably evolved from a rudimentary definition of people over the age of 65 who are dependent on others for activities of daily living and are often under institutional care (Rockwood et al. 1994). The current characterization of frailty is a geriatric syndrome characterized by age-associated decline in physiological reserve and function across multi-organ systems, leading to increased vulnerability for adverse health outcomes (Chen et al. 2014). The first frailty index was developed in 2004 by Rockwood et al. (Jones et al. 2004) and uses ongoing disease, physical and cognitive impairment as well as psychosocial risk factors that are age associated to assess the health state of an elderly person. Numerous similar models have since been developed and many have been found to have strong predictive validity for health outcomes in patients (Malmstrom et al. 2014). In critically ill patients, those who have higher frailty scores have been shown to have increased risk of adverse events, morbidity and mortality (Bagshaw et al. 2014).

In surgical research, frailty is increasingly used as an age-associated tool to assess vulnerability and has been found to be associated with poor clinical outcomes such as surgical site infections (Korol et al. 2013). A review of the orthopaedic literature reveals only one recently published study that demonstrates the use of a frailty index measure (Patel et al. 2014). The researchers found that patients aged 60 years or older with a hip fracture and a modified frailty score of 4 or higher had an increased risk of 1 and 2 year mortality (Patel et al. 2014). The importance of this study with reference to orthopaedic research has been underlined in an editorial that accompanied the publication of this study (Zampini 2014). The author of the editorial describes how significant physiological difference exists between older patients and the use of a frailty index allows us to objectively distinguish the different groups, which is important in order to improve orthopaedic clinical practice (Zampini 2014). This view is mirrored by recently published guidelines from the British Geriatric Society that recommend that older patients due to undergo surgical intervention should be assessed with the Edmonton Frail Scale as it may help with pre-operative optimization (Turner and Clegg 2014).

Our study demonstrates a wide range of definitions of age by chronology within hip fracture research. Subjective and variable population definitions in this field are likely to produce confusion for those attempting to interpret the evidence base to better inform their practice, and provide a realistic prognosis for their patients, as well as for organisations aiming to produce a health economic evaluation or a clinical practice guideline. The National Institute for Health Research in the United Kingdom (UK) produces policy guidance derived from health economic evaluations. An example of this is their health technology assessment comparing hemiarthroplasty and total hip replacement for intracapsular hip fracture patients (Carroll et al. 2011). Their conclusions are drawn from an analysis of 11 studies that include population definitions of elderly hip fracture patients that range from 50 to 70 years of age. The variability of age amongst these studies demonstrates that a more reliable and clinically relevant criteria are required for making clinical and funding decisions. These limitations have been recognized by health services, and there is growing opinion by policy makers that care pathways should be funded and delivered for elderly patients based on frailty index measures (NHS-England 2014).

In the United Kingdom (UK) hip fracture research is supported by the British Orthopaedic Association and the British Geriatric Society. Both groups are involved in the management of the National Hip Fracture Database (NHFD) which is used to enhance the quality of care and clinical outcomes for hip fracture patients (Sahota and Currie 2008). Although the NHFD in the UK does consider age in conjunction with comorbidity to produce case-mix adjusted outcomes, future orthopaedic research as well as national registry data should consider using frailty measures so that definitions of an elderly population are more systematic and uniform. Furthermore, focus on frailty measure rather than only chronological definitions of elderly age in orthopaedic research may provide frailty associated outcome data that are more applicable to clinical decision making in the care of elderly patients with different health states. Generic risk-assessment tools for surgery such as the American Society of Anesthesiologists’ (ASA) classification system (Wolters et al. 1996) and the P-POSSUM scoring system (Poon et al. 2005) have been invaluable in predicting patient outcomes and recognizing the variability of physiological reserve within a sub-group of older patients. The ability of frailty index measures to enhance the predictive power of the ASA system in older patients (Makary et al. 2010) underlines the need to use these tools in conjunction with each other in both clinical practice and research. In the case of the latter, sub-group differentiation of outcomes based on the use of such tools has the potential to inform clinical decision making through an evidence base that speaks to the broad range of health states within older patients.

### Study limitations

There are three limitations to this study. Firstly, by adopting a 1-year time horizon for our search it is likely that we limited the number of potential studies that could have been evaluated. Despite this we believe our search strategy produced a large enough and representative sample of orthopaedic studies to examine how elderly age is defined in recent orthopaedic literature.

Secondly, not all articles state intent to examine an intervention in an elderly population in their objective. The objective is often quite broad and such studies can draw conclusions regarding elderly patients from results that have performed sub-group analysis on different age groups. Although many of these studies were excluded from our analysis, it is unlikely that such studies would have different representative definitions of age by chronology. Furthermore, by only evaluating studies that stated an intention to examine an elderly population in their objective, there is increased focus on definitions that should be more robustly agreed by the authorship.

Finally, although a significant difference was not found in the range of reported definitions of an elderly person when examining the different research groups (region of development, orthopaedic sub-specialty and level of evidence), some populations within these groups were very small and therefore there is potential of type II error in our results.

## Conclusions

Orthopaedic research most commonly defines elderly age by chronological measures alone. Although persons who are 65 years of age were commonly described as elderly, this accounted for less than half of the studies we examined. Furthermore, there is considerable heterogeneity amongst these definitions of elderly age with an range of 50–80 years of age observed within the observed orthopaedic studies. These inconsistencies are not restricted to the region or country of research development, orthopaedic sub-specialty or the level of evidence of the article. Such variability is common amongst hip fracture research, an area in orthopaedics where a large proportion of patients are elderly and vulnerable. The findings of this study have widespread implications for orthopaedic healthcare policy and research. Health economic evaluations and clinical practice guidelines are likely to benefit from a homogenous and clinically relevant population description within the research they draw their recommendations from. Validated frailty index measures may provide an improved approach for defining elderly populations in orthopaedic research.
